# Effects of Immediate Coating on Unset Composite with Different Bonding Agents to Surface Hardness

**DOI:** 10.1055/s-0041-1740221

**Published:** 2022-02-18

**Authors:** Nantawan Krajangta, Supissara Ninbanjong, Sunisa Khosook, Kanjana Chaitontuak, Awiruth Klaisiri

**Affiliations:** 1 Department of Operative Dentistry, Faculty of Dentistry, Thammasat University, Pathumthani, Thailand; 2 Thammasat University Research Unit in Restorative and Esthetic Dentistry, Thammasat University, Pathumthani, Thailand; 3 Undergraduate Dental Student, Faculty of Dentistry, Thammasat University, Pathumthani, Thailand; 4 Private Practice, Kudchum Hospital, Yasothon, Thailand; 5 Private Practice, Kutchap Hospital, Udonthani, Thailand

**Keywords:** dental adhesive systems, microhardness, surface coating, thermocycling

## Abstract

**Objectives** This study evaluated the surface microhardness of composite, affected by surface coating with different dental adhesive systems.

**Materials and Methods** A total of 100 composite discs were divided into five groups. Group 1 was uncoated (control group C), and groups 2 to 5 were coated with different adhesive systems (OptiBond FL: FL, OptiBond SOLO Plus: SOLO, OptiBond XTR: XTR, and OptiBond All in one: AIO, respectively). The Vickers microhardness (VHN) was measured without and with 500 thermocycles.

**Statistical Analysis** The data were analyzed using two-way ANOVA and Tukey's posthoc test at the 95% confidence level.

**Results** At 24 hours, the VHN of C (59.96 ± 3.68) and FL (59.83 ± 4.54) were significantly higher than SOLO (51.73 ± 4.63) and AIO (51.45 ± 4.11). The VHN of XTR (54.96 ± 3.68) was not significant compared with that of C and all other groups. After thermocycling, VHN were significantly decreased in all groups. However, there were no significant differences among all groups.

**Conclusions** At 24 hours, composite coated with different adhesive systems have different effects to VHN. Thermocycling all adhesive resin systems coated on composite surface significantly decreased the VHN.

## Introduction

Dental composite was widely used in restorative dentistry and gained increasing popularity due to its pleasing aesthetic, minimal invasive of cavity preparation, remarkable mechanical properties' improvement, and decline in amalgam use, which was a cause for concern on account of mercury toxicity. 1 However, one of the drawbacks of dental composite was stickiness of material, due to the presence of viscous monomers. 1 Composite sticked to the instrument during insertion and condensation, causing difficulties in clinical handling and shaping to the anatomy of natural tooth. 1 2 3 Thus, the stickiness of composite increased the risk of poor adaptation, void and porosity formation. 4 To solve this problem, some clinicians used the dental adhesive to lubricate composite instrument or dental brush while shaping the smooth surface of composite. 5

Recently, Bisco Modeling Resin (Bisco Inc., Illinois, United States), Ultradent composite wetting resin (Ultradent Products Inc., Utah, United States), Brush and Sculpt resin (Cosmedent Inc., Illinois, United states.), and GC modeling liquid (GC Corp., Tokyo, Japan), G-Coat Plus (GC Corp.), Composite Primer (GC Corp.), and Modeling Resin (Kerr Corp., California, United States) were marketed specifically to use as sculpting composite or wetting agents, in order to make composite less sticky. 5 6 7 8 Nevertheless, the usage of dental adhesives is still preferred, because it does not require any additional material. 9 10 Many properties of composite affected by different adhesive lubrication between incremental filling have been evaluated, such as cohesive, 3 flexural and tensile strengths, 9 color and water solubilities. 9 Furthermore, the degree of conversion, translucency and color stabilities affected from adhesive coating on outer surface of composite have also been evaluated in previous studies. 11 12 However, the surface hardness of composite affected from different dental adhesive systems on the outer surface of composite has never been reported. In additions, composite in oral environment were subjected to temperature changes of beverages or foods. 6 9 10 Thermal stress, together with the presence of water, may lead to hydrolytic degradation of the interface between fillers and matrix of composite. Several studies have been reported the effects of thermocycling on the microhardness of composite restorations. 6 13 14 Surface microhardness was related to the degree of resin conversion, wear resistance, and long-term stability of composite. 6 10 13 14 15 16 Therefore, the objective of this study was to compare the surface microhardness of composite affected by surface coating with different dental adhesive systems at 24 hours and after 500 thermocycles.

## Materials and Methods

### Experimental Design and Specimen Preparation

A total of 100 specimens of disc-shaped composite (Harmonize, A 3.5E shade, Kerr, Orange, CA, United states) were prepared by placing in single increment, using stainless steel split mold (6-mm diameter, 2-mm thickness). The mold was placed on polyester matrix strip over a glass slab. A total of 100 specimens were divided into five groups ( *n* = 20). Group 1 was control, whereas groups 2 to 5 were coated with one layer of different types of dental adhesive used as surface coating.

1. Group C—Uncoated specimens were prepared as a control group,
2. Group FL—Coated specimens with adhesive resin of three-step etch and rinse (OptiBond FL (bonding bottle), Kerr, Orange, CA, United States).
3. Group SOLO—Coated specimens with adhesive resin of two-step etch and rinse (OptiBond SOLO Plus (primer and bonding bottle), Kerr, Orange, CA, United States).
4. Group XTR—Coated specimens with adhesive resin of two-step self-etch (OptiBond XTR [bonding bottle], Kerr) and
5. Group AIO—Coated specimens with adhesive resin of one-step self-etch (OptiBond All in one, Kerr)

**Fig. 1:**
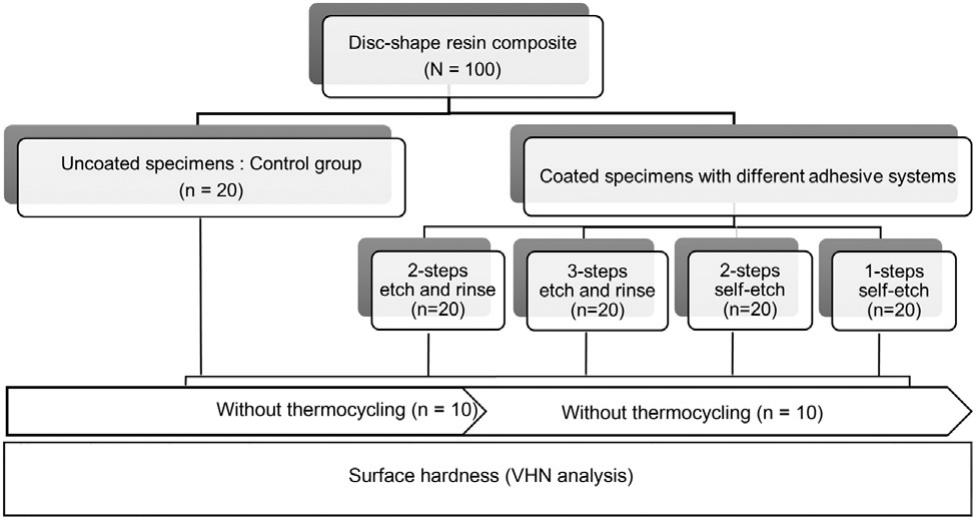
Experimental design of the study.

The name and compositions of composite and adhesive agents used in this experiment were presented in Table 1 and a schematic of the experimental design was given in Fig. 1 .

**Table 1:** The compositions of composite and adhesive agents used in this study

Product (Code name)	Material type	Compositions
Harmonize universal composite	Nonohybrid composite	Barium glass 400 nm, Silica and zirconia nanoparticles > 5nm. Average particle size 50 nm Total filler 81% w/w Bis-GMA, TEGDMA, Bis-EMA
Optibond FL (FL)	Three- step etch and rinse	Adhesive: Bis-GMA, HEMA, GDMA, CQ, ODMAB, fumed Silica, barium aluminum borosilicate glass, sodium hexafluorosilicate, coupling factor A174
Optibond SOLO Plus (SOLO)	Two-step etch and rinse	Adhesive: Bis-GMA, HEMA, GDMA, GPDM, water, ethanol, CQ, ODMAB, BHT, fumed Silica, barium aluminum borosilicate glass, sodium hexafluorosilicate, coupling factor A174
Optibond XTR (XTR)	Two-step self-etch	Adhesive: Ethanol, HEMA, MEHQ, CQ, fumed Silica, barium aluminum borosilicate glass, sodium hexafluorosilicate, coupling factor A174
Optibond All in one (AIO)	One-step self-etch	Adhesive: GPDM, HEMA, GDMA, Bis-GMA, water, ethanol, acetone, CQ, fumed Silica, barium aluminum borosilicate glass, sodium hexafluorosilicate, coupling factor A174

Abbreviations: A174, Gamma-methacryloxypropyltrimethoxysilane; BHT, 2,6-Di-(tert-butyl)-4-methylphenol; Bis-EMA, bisphenol A diglycidyl methacrylate ethoxylated; Bis-GMA, Bis-phenol-A-bis-(2-hydroxy-3-methacryloxypropyl) ether; CQ, camphorquinone; GDMA, Glycerol dimethacrylate; GPDM, Glycerophosphate-dimethacrylate; HEMA, 2-Hydroxyethylmethacrylate; MEHQ, 4-methoxyphenol or monoethyl ether hydroquinone; ODMAB, 2-(Ethylhexyl)-4-(dimetylamino)benzoate; TEGDMA, triethylene glycol dimethacrylate.

For control, after filling the composite into the mold, a polyester strip was covered the specimen; then, a glass slab was pressed over the matrix strip to obtain a flat surface.

For groups 2 to 5, after filling the composite into the mold, the surface was immediately coated with different adhesive systems by a single operator as follows:

1) The designated adhesive resin was dispensed into a clean dispenser; then, the instrument (CVIPC composite filling instrument, Hu-friedy, Chicago, United States) was dipped into the adhesive resin for a second. The excessive adhesive was removed by wiping at the edge of well dispenser for 3 seconds on each side of the instrument.
2) The surface of specimen was coated with designated adhesive resin using CVIPC to smoothen and flatten the surface.
3) A polyester matrix strip was covered the specimen; then, a glass slab was pressed over the matrix strip to obtain flat surface.

All of the specimens were polymerized from the top surface for 40 seconds with LED curing unit (SmartLite Focus, Densply Sirona, United Kingdom), and then gently removed from the split mold. The curing side of each specimen was polished using new aluminum oxide abrasive discs (OptiDisc series, Kerr, Orange, CA, United States) from medium to extrafine grit for 15 seconds in each step with water cooling every 2 seconds by the same operator. Each specimen was examined for flaws or voids under stereomicroscope

(X40) (Olympus SZX16, Hatagaya, Shibuya-ku, Tokyo, Japan). If any flaws or voids were presented, that sample must be excluded. Also, the final thickness after polishing were controlled to be 2 ± 0.5 mm. Then, all of the polished specimens were immersed in distilled water at 37 °C for 24 hours.

The specimens in each group were randomly divided into two subgroups ( *n* = 10): without and with thermocycling. In thermocycling group, the specimens were thermocycled in a thermocycle machine (Thermocycle machine Model TC301, King Mongkut's Institute of Technology, Bangkok, Thailand), according to ISO: 11405(2015) protocol of 500 cycles between temperatures of 5 and 55 °C with a dwell time of 20 seconds and a transfer time of 10 seconds.

### Surface Hardness Measurement

The surface Vickers microhardness (VHN) was measured on the polished surface of each specimen using a microhardness tester (FM-800, Future Tech, Kawasaki, Japan) with a 300 g load for 15 seconds. Three indentations per specimen were measured. The first indentation was located at the center of the specimen. The second and third indentations were located 1 mm apart from the first indentation at the right and left, according to ISO 6507–1(2018) 17 and ASTM E384–11(2011). 18 The VHN mean were calculated and recorded. VHN measurements were performed by one operator with good internal observer reliability (intraobserver reliability = 0.864)

### Statistical Analysis

The data were analyzed using IBM SPSS version 24.0 (SPSS Inc., Chicago, Illinois, United States). The normality of VHN was examined using the Shapiro–Wilk test. The Shapiro–Wilk test indicated normal distribution of VHN in all groups ( *p* > 0.05). The VHN average values were compared using two-way ANOVA and Tukey's posthoc test, considering two factors (dental adhesive type and thermocycling procedure) and their interaction. The statistical significance level was set at *p* < 0.05.

## Results

The mean VHN values and standard deviations (SD) for the composites were shown in Table 2 . Two-way ANOVA revealed that factors including dental adhesive system, thermocycling procedure and their interactions had statistically significant on VHN ( *p* < 0.01).

**Table 2:** VHN values for each group, without and with thermocycling

Aging process	Surface hardness values (VHN, kg/mm ^2^ ) (mean ± SD) of tested materials				
	Control	OptiBond FL	OptiBond SOLO plus	OptiBond XTR	OptiBond All in one
Without thermocycling	59.96 ± 3.68 ^Aa^	59.83 ± 4.54 ^Aa^	51.73 ± 4.63 ^BCa^	54.96 ± 3.68 ^ACa^	51.45 ± 4.11 ^BCa^
With thermocycling	53.57 ± 3.37 ^Ab^	44.24 ± 2.57 ^Bb^	41.81 ± 3.05 ^Bb^	43.76 ± 2.98 ^Bb^	40.95 ± 2.85 ^Bb^

Abbreviations: SD, standard deviation; VHN, Vickers microhardness.

* Note: Different letters (uppercase letter in the same row and lowercase letter in the same column) indicate statistically significant differences ( *p* < 0.05).

At 24 hours, without thermocycling, ranking of VHN were divided into two levels as shown in Table 2 : (1) control group (59.96 ± 3.68), Optibond FL (59.83 ± 4.54) and Optibond XTR (54.96 ± 3.68) and (2) Optibond XTR (54.96 ± 3.68), Optibond SOLO (51.73 ± 4.63) and Optibond AIO (51.45 ± 4.11). The VHN of Optibond XTR were not statistically significant compared with that of control and all other adhesives.

In contrast, after thermocycling, the VHN of all adhesive types were significantly lower than that of control. There was no statistical difference ( *p* > 0.05) in VHN among adhesive types.

Thermocycling significantly decreased the VHN of composite in both control and all type of adhesives ( *p* > 0.05).

## Discussion

Dental composite basically consisted of four main components: an organic polymer matrix, inorganic filler particles, a silane coupling agent that bound the filler particles with the matrix, and chemical groups that promote or modulate the polymerization. Monomers used in composite were base and diluent monomers. The base monomer, which has high viscosity, resulted in adherence of composite to the instrument during the application. Due to stickiness of composite, many techniques have been used to overcome this problem. Therefore, some clinicians used the dental adhesive to lubricate the instrument or dental brush while shaping the smooth surface of composite. 3 6 19 20

Previous studies on the effect of dental adhesive lubricated between incremental composite has found that there was no negative effect on cohesive strength of composite, 3 19 while it had negative effect on the diametral tensile strength and water uptake of composite. 21 If necessary, the adhesive solution of 3-step etch and hydrophobic rinse was recommended. 21 A study suggested that hydrophobic adhesive resin lubricated between incremental composite increased the physical and mechanical properties of material. The same trend was not presented with more hydrophilic adhesives. 9

There were some studies on the use of adhesive or modeling liquid coated on the outer surface of composite. A study showed that composite surface coated with adhesive has no alterations in color and opacity, and also enhanced the color stability to stain. 12 Other studies have shown that using modeling liquids on the outer surface could reduce the microhardness of composite. 6 7 22 However, the surface hardness of composite affected from different dental adhesive systems on the outer surface of composite has never been reported. Therefore, this *in vitro* study was emphasized on the effect of surface coating with different dental adhesive systems on microhardness of composite without and with 500 thermocycles.

With regard to the VHN of composite at 24 hours, without thermocycling, FL and XTR showed no difference compared with control. On the other hand, SOLO and AIO showed statistically significant lower VHN than control and FL. The different hydrophilicity of the adhesive resin compositions may lead to the different VHN of the composite. 23 Acidic primers and water-ethanol solvent were mixed with resin monomers in 2-step etch and rinse (SOLO) and 1-step self-etch (AIO). These adhesives were more hydrophilic than 3-step etch and rinse (FL) and 2-step self-etch (XTR), in which adhesive resin bottle was separated. 20 The hydrophilic adhesive of SOLO and AIO that coated on composite surface may decrease monomer conversion and polymerization than hydrophobic adhesive of FL and XTR. 16 On the other hand, hydrophobic adhesive resin of FL and XTR had no effect on surface hardness compared with control. 6 This phenomenon may be further explained, in that water in SOLO and AIO solvent left on composite surface and entrapped in composite before and during light cure process can lead to porous formation at the surface. Taken together, the decrease in monomer conversion and polymerization led to the reduction of surface hardness. 3 11 20 24

After 500 thermocycles, the VHN of composite was found decrease in all groups. Similar to many studies, thermocycling significantly decreased the microhardness of composite. 6 14 ISO TR 11450(2015) standards indicated that a thermocycling regimen comprising 500 cycles in water between 5 °C and 55 °C was appropriated for simulating artificial short-term aging of dental materials. 25 Organic matrix and inorganic fillers have different thermal expansion properties; therefore, they react differently to thermal changes. These could have an effect on VHN of composite. Moreover, water uptake of poorly polymerized resin in composite may accelerate hydrolysis degradation of matrix-filler interface in composite as well as induce superficial stress because of a high temperature gradient variation close to the surface. 14 After thermocycling, the results of this study showed that using the dental adhesive to lubricate composite instrument and coating on the composite surface significantly decreased the VHN than the control.

The effect of aging process on mechanical or physical properties of dimethacrylate resin depended on the chemical structure of resin. 16 26 Even though, at 24 hours, without thermocycling, adhesive layer of FL and XTR on composite surface has no effect on VHN. A small amount of low-viscosity adhesive resin probably penetrates into composite surface before curing process, making it more susceptible to water degradation and thermal stress after thermocycling. 14 27

From this study, hydrophobic FL and XTR can be coated minimally on composite surface, if necessary, because they have no negative influence on surface hardness of composite at 24 hours. Based on the observation by the authors, coating adhesive on composite surface can ease of the management and minimize the stickiness of the composite, corresponding with many previous studies. 3 19 However, ideally, the microhardness and other mechanical and physical properties of composite should not be deteriorated over time. As shown in this study, a significantly decreased in VHN after thermocycling may lead to an increase surface roughness after a period of use. 15 28 On the other hand, adhesive resin penetrating into the composite may prevent the occurrence of voids and defects inside the composite.

The disadvantage of coating adhesive on composite surface clinically might be mitigated by finishing and polishing steps. 6 29 However, the effect of the adhesive penetration into composite and finishing and polishing processes on microhardness is still unknown in this study. The limitations of this study were using only single type of composite and also single type of dental adhesive in each system (three-step etch and rinse, two-step etch and rinse, two-step self-etch and one-step self-etch). Further studies need to be tested with a variety of composites and dental adhesives.

## Conclusions

Composite coated with different adhesive systems have different effects on VHN at 24 hours. Hydrophobic adhesive resin (FL and XTR) have no negative influence on surface hardness of composite resin at 24 hours. After thermocycling, all of adhesive resin systems coated on composite surface significantly decreased the VHN.

## References

[1] Rasines Alcaraz M G Veitz-Keenan A Sahrmann P Schmidlin P R Davis D Iheozor-Ejiofor Z Direct composite resin fillings versus amalgam fillings for permanent or adult posterior teeth Cochrane Database Syst Rev 2014 03 CD005620 24683067 10.1002/14651858.CD005620.pub2

[2] Dunn W J Strong T C Effect of alcohol and unfilled resin in the incremental buildup of resin composite Quintessence Int 2007 38 01 e20 e26 17508071

[3] Perdigăo J Gomes G Effect of instrument lubricant on the cohesive strength of a hybrid resin composite Quintessence Int 2006 37 08 621 625 16922021

[4] Purk J H Dusevich V Glaros A Eick J D Adhesive analysis of voids in Class II composite resin restorations at the axial and gingival cavity walls restored under in vivo versus in vitro conditions Dent Mater 2007 23 07 871 877 16950506 10.1016/j.dental.2006.07.001 PMC1909915

[5] Liebenberg W H Bonding agent as an instrument lubricant: potential effect on marginal integrity Pract Periodontics Aesthet Dent 1999 11 04 475 476, 478 10635236

[6] Tuncer S Demirci M Tiryaki M Unlü N Uysal Ö The effect of a modeling resin and thermocycling on the surface hardness, roughness, and color of different resin composites J Esthet Restor Dent 2013 25 06 404 419 24172016 10.1111/jerd.12063

[7] Bayraktar E T Atali P Y Korkut B Kesimli E G Tarcin B Turkmen C Effect of modeling resins on microhardness of resin composites Eur J Dent 2021 15 03 481 487 34041724 10.1055/s-0041-1725577 PMC8382460

[8] Moghaddasi N Tavallali M Jafarpour D Ferooz R Bagheri R the effect of nanofilled resin-base coating on the mechanical and physical properties of resin composites Eur J Dent 2021 15 02 202 209 33111283 10.1055/s-0040-1716784 PMC8184272

[9] Münchow E A Sedrez-Porto J A Piva E Pereira-Cenci T Cenci M S Use of dental adhesives as modeler liquid of resin composites Dent Mater 2016 32 04 570 577 26850844 10.1016/j.dental.2016.01.002

[10] Sedrez-Porto J A Münchow E A Cenci M S Pereira-Cenci T Translucency and color stability of resin composite and dental adhesives as modeling liquids - A one-year evaluation Braz Oral Res 2017 31 e54 28678973 10.1590/1807-3107BOR-2017.vol31.0054

[11] Melo A MDS Santos T JSD Tertulino M D Degree of conversion, translucency and intrinsic color stability of composites during surface modeling with lubricants Braz J Oral Sci 2018 17 1 11

[12] Araujo F S Barros M CR Santana M LC Effects of adhesive used as modeling liquid on the stability of the color and opacity of composites J Esthet Restor Dent 2018 30 05 427 433 29607618 10.1111/jerd.12378

[13] Ghavami-Lahiji M Firouzmanesh M Bagheri H Jafarzadeh Kashi T S Razazpour F Behroozibakhsh M The effect of thermocycling on the degree of conversion and mechanical properties of a microhybrid dental resin composite Restor Dent Endod 2018 43 02 e26 29765905 10.5395/rde.2018.43.e26 PMC5952063

[14] Szczesio-Wlodarczyk A Sokolowski J Kleczewska J Bociong K Ageing of dental composites based on methacrylate resins—A Critical review of the causes and method of assessment Polymers (Basel) 2020 12 04 882 32290337 10.3390/polym12040882 PMC7240588

[15] Minami H Hori S Kurashige H Effects of thermal cycling on surface texture of restorative composite materials Dent Mater J 2007 26 03 316 322 17694738 10.4012/dmj.26.316

[16] Sideridou I D Karabela M M Bikiaris D N Aging studies of light cured dimethacrylate-based dental resins and a resin composite in water or ethanol/water Dent Mater 2007 23 09 1142 1149 17118438 10.1016/j.dental.2006.06.049

[17] ISO 6507–1 Metallic materials—Vickers hardness test—Part 1: Test method 2018

[18] ASTM E384–11, Standard test method for Knoop and Vickers hardness of materials 2011

[19] Barcellos D C Pucci C R Torres C R Goto E H Inocencio A C Effects of resinous monomers used in restorative dental modeling on the cohesive strength of composite resin J Adhes Dent 2008 10 05 351 354 19058680

[20] de Paula F C Valentin RdeS Borges B C Medeiros M C de Oliveira R F da Silva A O Effect of instrument lubricants on the surface degree of conversion and crosslinking density of nanocomposites J Esthet Restor Dent 2016 28 02 85 91 26865325 10.1111/jerd.12182

[21] Patel J Granger C Parker S Patel M The effect of instrument lubricant on the diametral tensile strength and water uptake of posterior composite restorative material J Dent 2017 56 33 38 27746333 10.1016/j.jdent.2016.10.006

[22] Kutuk Z B Erden E Aksahin D L Durak Z E Dulda A C Influence of modeling agents on the surface properties of an esthetic nano-hybrid composite Restor Dent Endod 2020 45 02 e13 32483531 10.5395/rde.2020.45.e13 PMC7239675

[23] Tay F R Pashley D H Have dentin adhesives become too hydrophilic? J Can Dent Assoc 2003 69 11 726 731 14653938

[24] Cadenaro M Breschi L Antoniolli F Degree of conversion of resin blends in relation to ethanol content and hydrophilicity Dent Mater 2008 24 09 1194 1200 18342363 10.1016/j.dental.2008.01.012

[25] ISO/TS 11405 Dentistry—Testing of adhesion to tooth structure 2015

[26] Delaviz Y Finer Y Santerre J P Biodegradation of resin composites and adhesives by oral bacteria and saliva: a rationale for new material designs that consider the clinical environment and treatment challenges Dent Mater 2014 30 01 16 32 24113132 10.1016/j.dental.2013.08.201

[27] Tay F R Carvalho R M Pashley D H Water movement across bonded dentin - too much of a good thing J Appl Oral Sci 2004 12(spe):12 25 10.1590/s1678-77572004000500003 20959943

[28] Drummond J L Degradation, fatigue, and failure of resin dental composite materials J Dent Res 2008 87 08 710 719 18650540 10.1177/154405910808700802 PMC2561305

[29] Pala K Tekçe N Tuncer S Serim M E Demirci M Evaluation of the surface hardness, roughness, gloss and color of composites after different finishing/polishing treatments and thermocycling using a multitechnique approach Dent Mater J 2016 35 02 278 289 27041019 10.4012/dmj.2015-260

